# Partial oral antibiotic treatment for bacterial brain abscess: an open-label randomized non-inferiority trial (ORAL)

**DOI:** 10.1186/s13063-021-05783-8

**Published:** 2021-11-12

**Authors:** Jacob Bodilsen, Matthijs C. Brouwer, Diederik van de Beek, Pierre Tattevin, Steven Tong, Pontus Naucler, Henrik Nielsen

**Affiliations:** 1 Department of Infectious Diseases, Aalborg University Hospital, Mølleparkvej 4, 9000 Aalborg, Denmark; 2 European Society for Clinical Microbiology and Infectious Diseases Study Group of Infections in the Brain (ESCMID), Basel, Switzerland; 3 Department of Neurology, Amsterdam Neuroscience, Amsterdam UMC, University of Amsterdam, Amsterdam, The Netherlands; 4 Department of Infectious Diseases and Intensive Care Unit, Pontchaillou University Hospital, Rennes, France; 5 Réseau National de Recherche Clinique en Infectiologie (RENARCI), Paris, France; 6 Victorian Infectious Diseases Service, The Royal Melbourne Hospital, Parkville, Australia; 7 Department of Infectious Diseases University of Melbourne, at the Peter Doherty Institute for Infection and Immunity, Melbourne, Victoria Australia; 8 Department of Infectious Diseases, Karolinska University Hospital, Stockholm, Sweden; 9 Department of Infectious Diseases and Department of Clinical Medicine, Aalborg University Hospital, Aalborg, Denmark

**Keywords:** Brain abscess, Cerebral abscess, Treatment, Randomized controlled trial, Non-inferiority, Antibiotics, Oral, Intravenous

## Abstract

**Background:**

The advised standard treatment for bacterial brain abscess following surgery is 6 to 8 weeks of intravenous (IV) antibiotic treatment, but an early switch to oral antibiotic treatment has been suggested to be equally effective.

**Methods:**

This investigator-initiated, international, multi-center, parallel group, open-label, randomized (1:1 allocation) controlled trial will examine if oral treatment after 2 weeks of IV antibiotic therapy is non-inferior to standard 6–8 weeks of IV antibiotics for bacterial brain abscess in adults (≥ 18 years of age). The study will be conducted at hospitals across Denmark, the Netherlands, France, Australia, and Sweden. Exclusion criteria are severe immunocompromise or impaired gastro-intestinal absorption, pregnancy, device-related brain abscesses, and brain abscess caused by nocardia, tuberculosis, or *Pseudomonas spp.* The primary objective is a composite endpoint at 6 months after randomization consisting of all-cause mortality, intraventricular rupture of brain abscess, unplanned re-aspiration or excision of brain abscess, relapse, or recurrence. The primary endpoint will be adjudicated by an independent blinded endpoint committee. Secondary outcomes include extended Glasgow Outcome Scale scores and all-cause mortality at end of treatment as well as 3, 6, and 12 months since randomization, completion of assigned treatment, IV catheter associated complications, durations of admission and antibiotic treatment, severe adverse events, quality of life scores, and cognitive evaluations. The planned sample size is 450 patients for a one-sided alpha of 0.025 and a power of 90% to exclude a difference in favor of standard treatment of more than 10%. Date of initiation of first study center was November 3, 2020, with active recruitment for 3 years and follow-up for 1 year of all patients.

**Discussion:**

The results of this study may guide future recommendations for treatment of bacterial brain abscess. If early transition to oral antibiotics proves non-inferior to standard IV treatment, this will provide considerable health and costs benefits.

**Trial registration:**

ClinicalTrials.gov NCT04140903, first registered 28.10.2019. EudraCT number: 2019-002845-39, first registered 03.07.2019

## Administrative information

Note: the numbers in curly brackets in this protocol refer to SPIRIT checklist item numbers. The order of the items has been modified to group similar items (see http://www.equator-network.org/reporting-guidelines/spirit-2013-statement-defining-standard-protocol-items-for-clinical-trials/).

**Table:** 

Title {1}	Partial oral antibiotic treatment for bacterial brain abscess: An open-label randomized non-inferiority trial (ORAL)
Trial registration {2a and 2b}.	ClinicalTrials.gov identifier: NCT04140903, first registered 28.10.2019. EudraCT number: **2019-002845-39**, first registered 03.07.2019
Protocol version {3} ^1^	Version 1.2 of March 10, 2021
Funding {4}	Economic support for all aspects of conducting the trial is provided by the Novo Nordisk Foundation (Grant 0057510)
Author details {5a}	Jacob Bodilsen (Aalborg University Hospital, Denmark) Henrik Nielsen (Aalborg University Hospital, Denmark) Matthijs C. Brouwer (Amsterdam UMC, The Netherlands) Diederik van de Beek (Amsterdam UMC, The Netherlands) Pierre Tattevin (Rennes University Hospital, France) Steven Tong (Royal Melbourne Hospital, Australia) Pontus Naucler (Karolinska, Stockholm, Sweden)
Name and contact information for the trial sponsor {5b}	Henrik Nielsen, Professor Department of Infectious Diseases, Aalborg University Hospital Mølleparkvej 4, 9000 Aalborg, Denmark henrik.nielsen@rn.dk + 45 97663920
Role of sponsor {5c}	The sponsor (HN) and the steering committee (JB, HN, MCB, DvdB, ST, PN, PT) will be responsible for trial completion, data analysis and interpretation.

## Introduction

### Background and rationale {6a}

Brain abscess is a serious infection with a considerable impact on patients’ lives [1–5]. Although it is a rare disease, the incidence has been increasing in the past 30 years with rates of 0.9/100,000/year corresponding to 6700 cases per year in Europe [6, 7]. Recent studies suggest 30-day, 90-day, and 1-year mortality rates of 7–11, 13, and 19–20% [6, 8]. Risk factors for mortality in brain abscess patients are advanced age and certain comorbidities such as immunocompromise or congenital heart disease [6, 9]. Sequelae occur frequently in surviving patients, and the total rate of unfavorable outcome defined as a Glasgow Outcome Scale (GOS) score < 5 was found to be 32% (95% CI 21–44) after 6 months since discharge based on unpublished data from the nationwide prospective clinical database of the Danish Study Group of Infections of the Brain (DASGIB) in years 2015 to 2018 [10, 11].

Treatment remains a challenge because of the limited penetration of antibiotics into the abscess due to the blood-brain-barrier and limited surgical options to completely remove the abscess [12]. The standard treatment approach usually consists of a combination of neurosurgical aspiration of the abscess and prolonged high-dose antibiotic therapy to ensure eradication of bacteria within the abscess cavity. There are no randomized controlled trials on antibiotic treatment of brain abscess to evaluate the standard regimen in immunocompetent individuals of intravenous (IV) 3rd generation cephalosporin and metronidazole for 6–8 weeks [3, 12]. However, other regimens have been suggested in the literature as well, including additional 6–12 weeks of oral antibiotics following the intravenous treatment, or shorter intravenous antibiotic courses when surgical evacuation has been performed [2, 12, 13]. Intraventricular rupture of brain abscess (IVROBA), severe immunocompromise, and certain pathogens (e.g., mycobacteria, nocardia, and fungi) may on the other hand require extended treatment for several months up to years [12].

Reports of treatment failure, relapse, and recurrence are rare with current treatment recommendations [8, 12, 13]. However, the long duration of IV treatment is often strenuous for patients to endure with associated discomfort of long-term admission, risks of hospital-acquired infections, and IV catheter complications (infection, bleeding, venous thrombosis, and need for IV catheter replacement due to malfunction). In addition, costs associated with long admissions are significant. Some hospitals may offer outpatient parenteral antibiotics to selected patients, but logistic demands for this option are also high and education of patients and staff is needed to ensure adherence to therapy, appropriate monitoring of efficacy and safety, social support, and easy access to medical counsel.

Shortened IV treatment for bacterial brain abscess has been a controversial issue since the Infection in Neurosurgery Working Party of the British Society for Antimicrobial Chemotherapy recommended 1–2 weeks of IV therapy in patients with a good clinical response followed by an appropriate oral regimen [12, 14]. Although prone to selection and publication bias, this regimen has been reported to be safe and effective in a few retrospective observational studies with a total of 231 patients of which 18 (8%) died [15–19]. Moreover, two recent non-inferiority trials of early transition to oral treatment for endocarditis and bone and joint infections showed that an early switch to oral therapy in serious and invasive infections can be as effective as IV treatment, opening up the possibility this may also be feasible for brain abscess patients [20, 21]. A post hoc analysis of patients with endocarditis showed a decreased mortality in patients assigned to shortened IV therapy compared with standard therapy (16% vs. 27%, hazard rate ratio 0.57 [95% CI 0.37–0.87]) after a median of 3.5 years of observation [22]. In addition, oral treatment has been considered standard of care for other focal central nervous system (CNS) infections such as cerebral toxoplasmosis and CNS tuberculomas for decades. However, properly controlled trials are needed to examine this treatment strategy in bacterial brain abscess patients.

### Objectives {7}

**Fig. 1:**
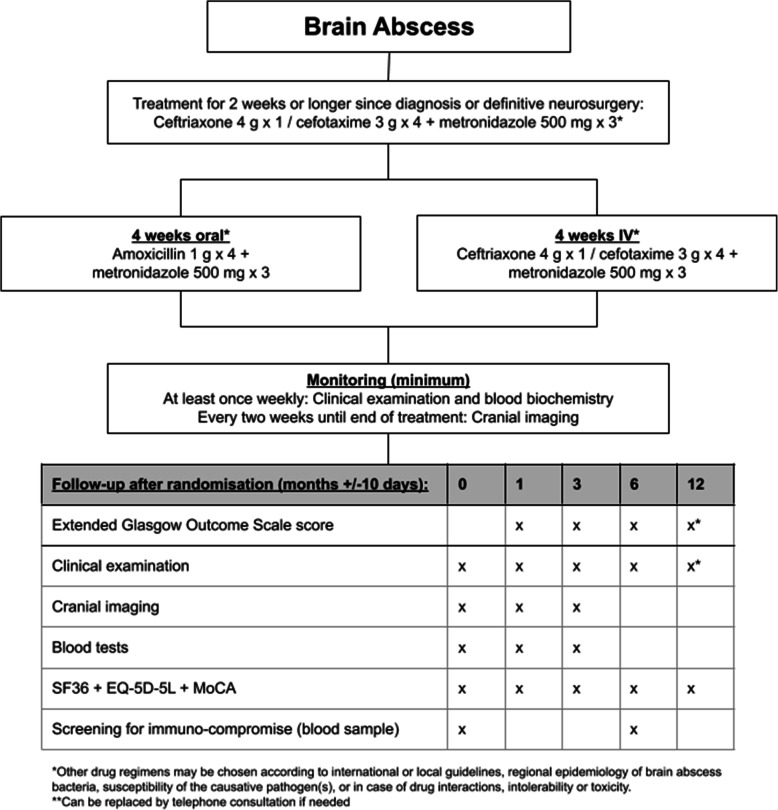
General overview of the trial

To investigate if early transition to oral treatment after 2 weeks or longer of IV antibiotic therapy is non-inferior to standard 6 weeks or longer of IV antibiotic treatment for bacterial brain abscess (Fig. 1).

### Trial design {8}

Investigator-initiated, international, multi-center, parallel group, open-label, randomized (1:1 allocation), controlled, non-inferiority trial.

## Methods: participants, interventions, and outcomes

### Study setting {9}

**Fig. 2:**
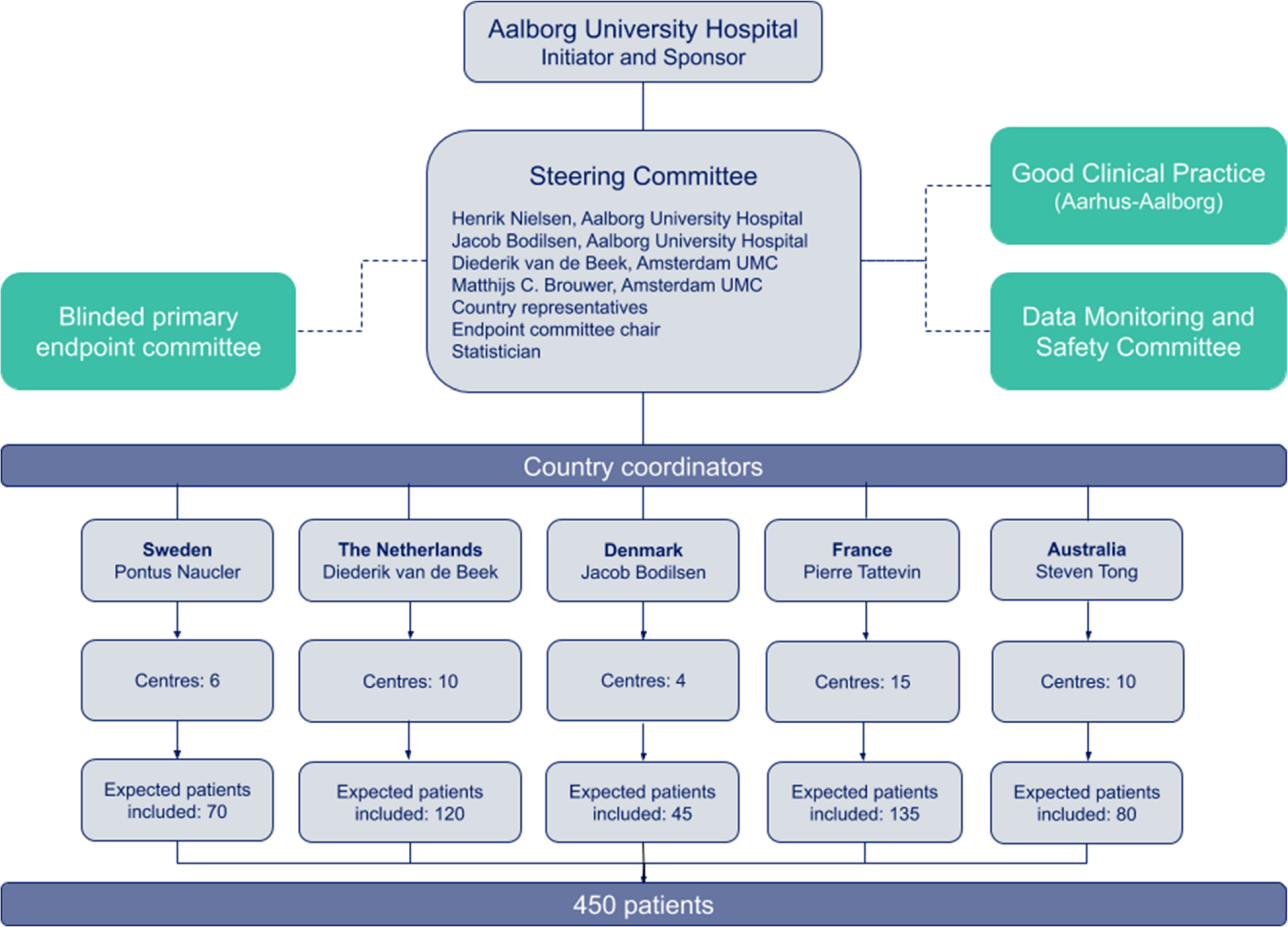
Organisation of the ORAL trial

The study will be conducted at hospitals in Denmark, the Netherlands, France, Australia, and Sweden (Fig. 2)

### Eligibility criteria {10}

All patients admitted at participating centers with brain abscess will be assessed for eligibility by the local study investigator or research nurse. Each local study investigator is responsible for assigning patients to their randomized treatments.

#### Inclusion criteria

Adults ≥ 18 years of age with bacterial brain abscess defined as:

1. A clinical presentation (e.g., headache, neurological deficit or fever) and cranial imaging (CT or MRI) consistent with brain abscess according to the hospital radiologist AND
2. The physician responsible for the patient decides to treat the patient for bacterial brain abscess

Further requirements for inclusion are:

3. Ability to absorb oral medication (including by nasogastric tube) and availability of IV access.
4. To have received empiric or targeted (according to in vitro susceptibility) IV antibiotic therapy for bacterial brain abscess for 14 consecutive days or longer before randomization and no additional aspiration or excision of brain abscess anticipated
5. Expected to be treated with antibiotic therapy for at least another 14 days after time of randomization
6. No progression in neurological deficits or occurrence of new-onset neurological symptoms (excluding seizures) within 5 days before time of randomization

#### Exclusion criteria

Patients fulfilling any of the following criteria will be excluded:

1. Hypersensitivity to an antibiotic intended for use in the patient and no alternative drugs available;
2. Expected substantially reduced compliance with treatment (e.g., IV drug abuse);
3. Pregnancy (proven by positive urine or plasma human chorionic gonadotropin test in fertile women);
4. Lactating women;
5. Concomitant treatment for proven or suspected CNS infection caused by mycobacteria, *Nocardia spp.*, *Pseudomonas spp.*, fungi, toxoplasmosis, or other CNS parasites;
6. Device-related brain abscesses (e.g., deep brain stimulators, ventriculo-peritoneal shunts);
7. Severe immunocompromise defined as ongoing need for biological- or chemotherapy, prednisolone > 20 mg/day for ≥ 14 days, uncontrolled HIV/AIDS (see Glossary), hematological malignancies (see Glossary), and organ transplant recipients;
8. Concomitant or unrelated infections necessitating IV antibiotics beyond seven days of duration after time of randomization;
9. Nosocomial brain abscess;
10. Previous enrolment into this trial;
11. Patients not capable of providing informed consent at time of randomization.

### Who will take informed consent? {26a}

Patients will be approached by the local study investigator or specially trained research nurse and asked to participate in the study after 10 to 14 days of appropriate brain abscess treatment. They will be encouraged to bring relatives to the meeting. Patients will be given oral and written information of their rights as study participants and of the possibility to withdraw from the study at any time without consequences for further treatment and care.

### Additional consent provisions for collection and use of participant data and biological specimens {26b}

We will also examine patients for Hereditary Hemorrhagic Telangiectasia by mutations in the genes encoding endoglin and the Activin A receptor type II-like 1 [23–25]. For this substudy, a separate patient consent form will be obtained and blood samples drawn at time of randomization and after 6 months since randomization.

## Interventions

### Explanation for the choice of comparators {6b}

Early switch to oral antibiotics may decrease risks of complications to treatment and nosocomial infections as well as be a more a cost-effective treatment with increased convenience for patients and hospitals alike.

### Intervention description {11a}

**Fig. 3:**
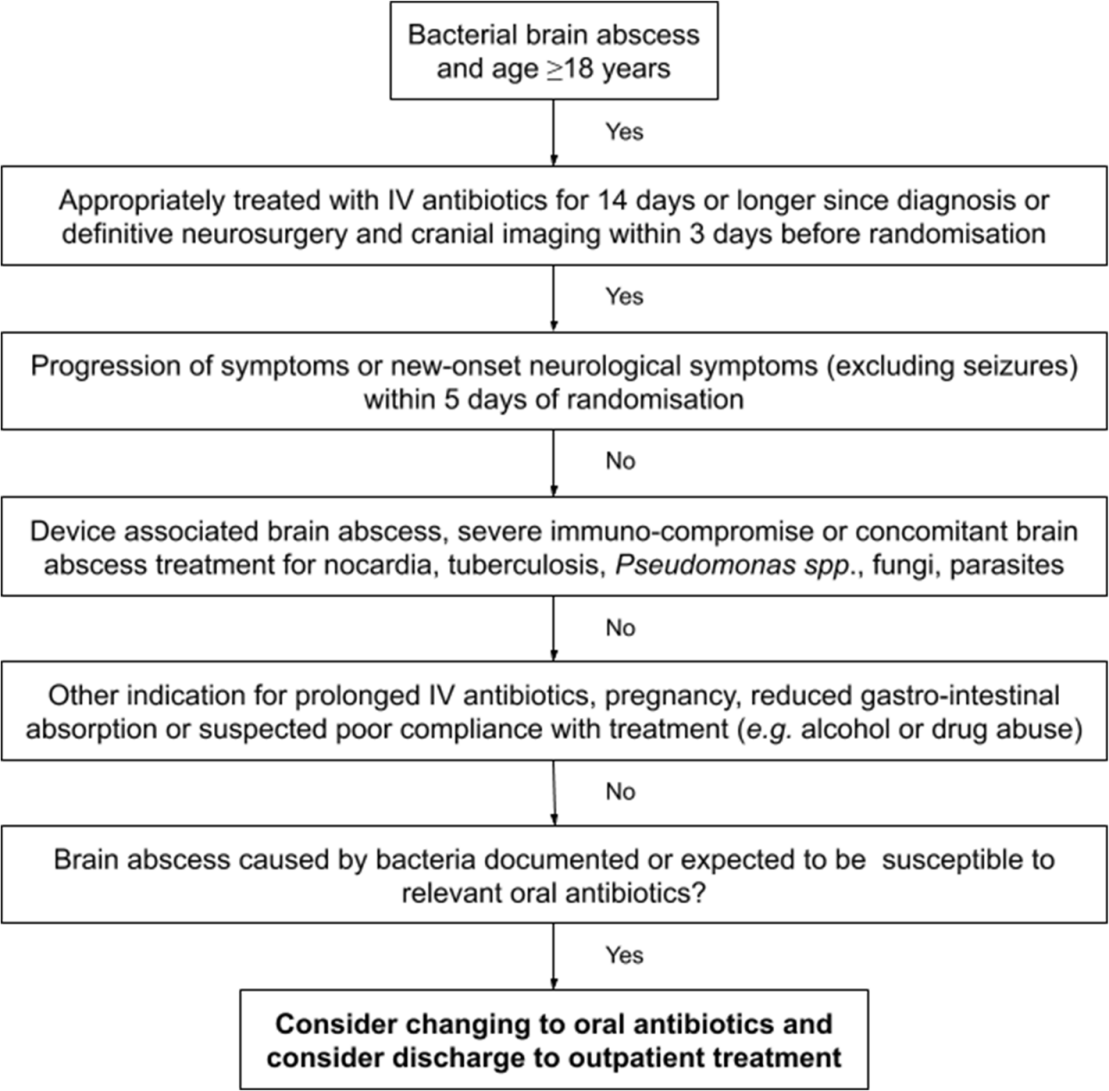
Flowchart of patient screening and inclusion

Patients will be randomized to early switch to oral antibiotic therapy (Table 1) or continuation of standard IV therapy for bacterial brain abscess for the remaining duration of treatment. Randomization will take place after 2 weeks or longer of appropriate antibiotic therapy for bacterial brain abscess since definitive neurosurgery of brain abscess or, in case of no planned diagnostic neurosurgical procedure, since initiation of intravenous antibiotics recommended for bacterial brain abscess by international or local guidelines (Fig. 3) [3, 12].

**Table 1:** Antibiotic treatment recommendations for the ORAL trial^a^

	Intervention group (2 weeks IV + 4 weeks oral)	Standard group (6 weeks IV)
**First 2 weeks**	Ceftriaxone 4 g × 1 / cefotaxime 3 g × 4 + metronidazole 500 mg × 3	Ceftriaxone 4 g × 1 / cefotaxime 3 g × 4 + metronidazole 500 mg × 3
**Next 4 weeks**	Oral amoxicillin 1 g × 4 + metronidazole 500 mg × 3	Ceftriaxone 4 g × 1 / cefotaxime 3 g × 4 + metronidazole^b^ 500 mg × 3
In case of *Streptococcal spp.* with a minimal inhibitory concentration for penicillin ≥1 mg/L, beta-lactam allergy, non-susceptibility, interaction with other drugs, or development of drug fever.	a) Oral moxifloxacin 400 mg × 1 + metronidazole 500 mg × 3 b) Oral linezolid 600 mg × 2 + metronidazole 500 mg × 3 c) Oral clindamycin 600 mg × 4	a) Meropenem 2 g × 3 b) Moxifloxacin 400 mg × 1 + metronidazole 500 mg × 3 c) Clindamycin 600 mx 4

^a^Other drug regimens may be chosen by the local study investigator at each site according to international or local guidelines, regional epidemiology of brain abscess bacteria, susceptibility of the causative pathogen(s), renal or liver impairment, or in case of drug interactions, intolerability or toxicity. Such changes should be consulted with infectious disease specialists and/or clinical microbiologist taking into consideration the pharmacokinetic/pharmacodynamic properties of the chosen antibiotics

^b^Can be replaced by oral metronidazole and may be stopped earlier during treatment to limit risk of side effects at the discretion of the treating physician

Definitive neurosurgery is defined as last aspiration or excision of brain abscess based on all available clinical information without any further expected aspirations or excisions of brain abscess at time of randomization. Cranial imaging is required within 3 days and blood tests (c-reactive protein, complete blood count, creatinine, sodium, potassium, bilirubin, and aspartate transaminase) within 1 day before time of randomization for documentation of subsequent cure or treatment failure.

### Criteria for discontinuing or modifying allocated interventions {11b}

Patients will discontinue the study if bacterial brain abscess is disproved, if they experience a primary outcome, or if the randomly assigned antibiotic strategy (oral or IV) is otherwise considered to be clinically unacceptable and incompatible with good clinical care. If a patient experiences a sudden unexpected serious adverse reaction (SUSAR), the trial intervention in this patient will also be stopped. All participants have the right to withdraw from the intervention at any time without any explanation and receive routine clinical care. However, all study participants will remain in the study for follow-up and will be included in the intention-to-treat analysis.

### Strategies to improve adherence to interventions {11c}

Morisky scores will be used to assess adherence to oral antibiotic treatment at each outpatient contact. Moreover, the trial will be externally monitored according to a predefined template developed in collaboration with the Good Clinical Practice (GCP) unit in Aarhus-Aalborg (Denmark).

### Relevant concomitant care permitted or prohibited during the trial {11d}

Patients randomized to early switch to oral antibiotic therapy will, if needed, be allowed a short course (≤ 7 days) of IV antibiotic therapy for unrelated infections during oral treatment. Patients in both treatment arms are allowed to be discharged to outpatient monitoring during the study. Other interventions for brain abscess as part of routine care as well as for other medical conditions are also allowed during the study.

### Provisions for post-trial care {30}

Study participants in Denmark will be insured by the Patient Insurance Association and protected by the “Patients’ Rights Act” and “Act on Patient Safety in the Danish Health Care System.” Similar insurance and health care legislature will also apply or be obtained for patients from other participating countries.

### Outcomes {12}

Brain abscess is a serious and potentially fatal condition. Besides death, important aspects of treatment failure for both patients and physicians include risk of IVROBA with an associated in-hospital mortality of 27–85% and frequent need of intraventricular drainage [3], numbers requiring re-aspiration or excision as well as risks of relapse or recurrence.

In addition, a measure of functional outcome of patients is crucial. The Extended Glasgow Outcome Scale (E-GOS) score was developed to assess the functional outcome of patients with traumatic brain injury and has since been widely applied in studies of CNS infections [11, 26–28]. The score reflects both measurable physical and cognitive deficit as well as more intangible impairment that nonetheless greatly impact the social functioning of patients [26]. In addition to validated standardized interviews developed for use in admitted patients or in outpatient settings, the score has also been shown to maintain very high reliability for use in telephone interviews (weighted K-statistic of 0.92 between in-person and telephone interview and 0.84 for inter-rater reliability) and as postal questionnaires [29–31]. A validated French translation of the structured interview is also available for use [32].

#### Primary endpoint

The primary outcome is 6-month risk of treatment failure defined as a composite of either death, IVROBA, unplanned (re-)aspiration or excision of brain abscess, relapse, or recurrence (Table 2).

**Table 2:** Definition of the primary composite endpoint of treatment failure at 6 months since randomization

Component	Definition
**All-cause mortality**	All deaths observed among study participants within 6 months since randomization.
**IVROBA**	Sudden serious clinical deterioration and cranial imaging suggesting intraventricular rupture of brain abscess after time of randomization.
**Unplanned neurosurgery**	Unplanned (re-)aspiration or excision of brain abscess after randomization. This does not include, e.g., planned insertion of ventriculo-peritoneal drain or corrective surgery of dural leak.
**Relapse**	a. Cranial imaging showing an increase in brain abscess volume (height × depth × width/2) by 20% compared with maximum volume at time of randomization or formation of additional brain abscesses after randomization. b. Clinical deterioration attributable to treatment failure of brain abscess. This does not include clinical deterioration caused by unrelated infections or other medical conditions^a^. c. The local investigator assesses that clinical cure cannot be obtained without IV antibiotics after 4 weeks *or longer* of oral antibiotics^b^. This does not include remnant cystic formation at location of brain abscess or residual enhancement on cranial imaging of an otherwise asymptomatic patient [33–36].
**Recurrence**	New brain abscess formation after end of antibiotic treatment

^a^ Patients randomized to early switch to oral antibiotic therapy will, if needed, be allowed a short course (≤ 7 days) of IV antibiotic therapy for unrelated infections during oral treatment

^b^Patients in both treatment arms may sometimes require prolonged treatment and complete radiological resolution of brain abscess often lags behind clinical cure. As long as the clinical condition improves, this is not considered treatment failure and patients can continue the assigned treatment strategy until clinical cure

An independent blinded endpoint committee member from each country will adjudicate every non-fatal primary outcome by review of case report forms and medical records redacted for all information on allocated treatment and IV catheter insertion. A blinded neuroradiologist will adjudicate all cranial imaging results in patients assessed to experience IVROBA, relapse, or recurrence.

#### Secondary endpoints

- Occurrence of each component of the composite primary endpoint after 6 months since randomization
- Unfavorable outcome (E-GOS < 7) and all-cause mortality at end of treatment as well as 3, 6, and 12 months since randomization
- Unfavorable outcome at 6 months since randomization using sliding dichotomy of E-GOS stratified by Charlson Comorbidity Index (CCI) scores at time of randomization [37]
- Completion and adherence to assigned treatment strategy (Morisky scores)
- IV catheter related complications (bleeding, infection, venous thrombosis, need for replacement) during brain abscess treatment
- Durations of admission and antibiotic treatment for brain abscess
- Number of readmissions within 6 months since randomization
- Occurrence of *Clostridioides difficile* associated diarrhea during brain abscess treatment
- Edema on cranial imaging at 3 months since randomization.
- Severe adverse events during brain abscess treatment
- Quality of life scores and cognitive evaluations (SF-36, EQ-5D-5L, MoCA) at time of randomization, end of treatment and 3, 6, and 12 months since randomization

### Participant timeline {13}

Table 3 shows the participant timeline.

**Table 3:** Participant timeline

	Study period							
	Enrolment	Allocation^a^	Monitoring during treatment		End of treatment	Follow-up since randomization (months)		
Study phase	Admission to randomization		Weekly	Bi-weekly		3	6	12^b^
Enrolment								
Eligibility screen	✓							
Informed consent	✓							
Cranial imaging	− 3 to 0 days							
Allocation		✓						
Intervention		●-------------------------------------------------------------●						
Assessments								
E-GOS					✓	✓	✓	✓
Clinical examination		✓	✓		✓	✓	✓	✓
Cranial imaging				✓	✓	✓	✓	
Morisky scores (oral arm)			✓					
Blood tests		✓	✓		✓	✓	✓	
SF-36 + EQ-5D-5L + MoCA		✓			✓	✓	✓	✓
Screening for immunodeficiency		✓					✓	

*E-GOS* Extended Glasgow Outcome Scale score. Blood tests include c-reactive protein, complete blood count, creatinine, liver function tests

^a^After 14 days or longer since definitive neurosurgical treatment or IV antibiotic therapy for brain abscess

^b^Can be replaced by telephone consultation

### Sample size {14}

We will use a non-inferiority design to determine if early switch to oral antibiotics is inferior to standard treatment with IV antibiotics for 6 weeks in brain abscess patients by a predefined margin or more (null hypothesis). For non-inferiority studies of hospitalized pneumonia patients, the United States Food and Drug Administration have previously suggested an absolute risk difference in mortality of 10% [38].

Brain abscess is a rare condition and detailed information on the short- and long-term risks of complications or death during the course of treatment is sparse. Besides previously published studies, we primarily used unpublished data from an ongoing nationwide prospective observational Danish database (DASGIB) to assess the risk of each component in the primary composite endpoint [10, 11]. For patients alive 2 weeks after admission for brain abscess, the estimated 6-month mortality is 5–7%, risk of IVROBA is 1%, unplanned (re-)aspiration or excision is 2–4%, and risk of recurrence is 0–1%. Thus, we assume that the primary endpoint will occur in 11% (range 8–13%) of patients in both groups after randomization.

Long-term IV antibiotic treatment is associated with increased duration of admission and risks of hospital-acquired infections, psychological strain, large economic expenses, and perhaps even increased mortality [22]. Based on all these considerations, we chose a margin of 10% to be the largest acceptable difference in absolute risk between groups.

By setting *α* to 0.025 (one-sided) and *β* to 0.1, we need to recruit 2 × 206 patients (total 412 patients) to be 90% certain, that the upper limit of a one-sided 97.5% CI (or equivalently a two-sided 95% CI) will exclude a difference in favor of standard treatment of more than 10%. To account for potential loss to follow-up, cross-over (from oral to IV therapy), and withdrawals, we aim to include 2 × 225 patients (total 450 patients).

### Recruitment {15}

Time of randomization at 2 weeks after definitive aspiration/excision or since start of antibiotic treatment for brain abscess limits missed study inclusion due to acuteness of disease. Incentives for patient recruitment include a pragmatic electronic case report form and a predefined publication strategy.

**Fig. 4:**
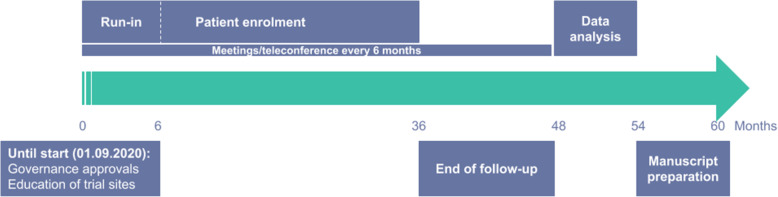
Patient timeline

The study will begin with a run-in period of 6 months in selected centers before opening up for study inclusion in other hospitals. We expect to be able to include one third to half of all brain abscess patients in participating centers yielding approximately 150 enrolled patients each year. Thus, we anticipate patient inclusion to be completed (*n* = 450) within 3 years since start of the study (Fig. 4).

## Assignment of interventions: allocation

### Sequence generation {16a + 16b + 16c}

We will use a centralized internet-based computer-generated randomization schedule prepared and overseen by an experienced statistician. Patients will be randomized in a 1:1 ratio in permuted blocks of 2 to 6 and stratified by country and largest brain abscess diameter over or under 3 cm.

### Concealment mechanism {16b}

The randomization schedule will be unavailable to those who enroll participants or assign interventions. Patients will be randomized directly within the electronic case report form (REDcap) after obtainment of patient consent.

### Implementation {16c}

The allocation sequence is computed-generated and overseen by the trial statistician. Local investigators or study nurses will enroll patients, while local investigators are responsible for assigning patients to the interventions.

## Assignment of interventions: Blinding

### Who will be blinded {17a}

An independent blinded endpoint committee member from each country will adjudicate every non-fatal primary outcome by review of case report forms and medical records redacted for all information on allocated treatment and IV catheter insertion. A blinded neuroradiologist will adjudicate all cranial imaging results in patients assessed to experience IVROBA, relapse, or recurrence.

### Procedure for unblinding if needed {17b}

Local investigators responsible for management of the individual patients are not blinded to treatment.

## Data collection and management

### Plans for assessment and collection of outcomes {18a}

Prior to study onset at each site, local study staff will receive a series of standardized operating procedures on how to screen and randomize patients, obtain and enter study data, specific procedures related to the study (e.g., obtainment of blood samples for immune-genetic analyses), reporting of potential primary outcomes in need of adjudication (i.e., non-fatal primary outcomes) to the Sponsor and blinded endpoint committee members, and how to use the included questionnaires (e.g., E-GOS, SF-36, and Morisky scores).

During admission, the local study investigator will record information in a case report form using the internet-based REDcap the electronic data capture tools hosted at North Denmark Region. Data will include:

- Baseline demographics at time of randomization (age, sex, residence, contact information), comorbidity including history of smoking and alcohol abuse, vital signs, clinical status and results of physical examinations, microbiological findings (including susceptibility patterns of the causative pathogens), and ancillary radiological investigations.
- Predisposing condition(s) and any concomitant foci of infection
- Results of cranial imaging (within 3 days) and routine blood tests (i.e., c-reactive protein, complete blood count, liver function tests, sodium, potassium, and se-creatinine) at time of randomization.
- Duration of hospital stay
- Death
- IVROBA
- Neurosurgical procedures for brain abscess before and after randomization
- Relapse
- Change of antibiotic treatment (drug, dosages) and/or strategy (iv vs. oral)
- Duration of antibiotic treatment for brain abscess
- IV and oral antibiotic treatment for unrelated infections
- IV catheter associated complications (bleeding, infection, venous thrombosis, need for replacement)
- Adherence to oral antibiotics (Morisky scores)
- Seizures (self-reported and witnessed)
- Serious adverse events (SAEs)
- Adverse drug reactions

Included variables at follow-up visits after randomization (3, 6, and 12 months after randomization):

- Mortality
- Relapse or need of change from oral to IV antibiotics
- Recurrence of brain abscess
- E-GOS scores (structured interviews)
- SAEs
- Admissions (reason, results of any cranial imaging)
- Seizures (self-reported and witnessed)
- Routine biomarkers (c-reactive protein, complete blood count, liver function tests)
- Quality of life and cognitive scores (SF-36, EQ-5D-5L, MoCA)
- Follow-up time

The E-GOS is frequently used for CNS infections and has a high reliability by interview by either physical meetings, telephone, or as postal questionnaire [27]. The Morisky score is a validated instrument of medication adherence in an outpatient setting and a score > 2 indicates low adherence, 1 or 2 indicates medium adherence, and a score of 0 equals high adherence [39, 40].

### Plans to promote participant retention and complete follow-up {18b}

In case a patient is transferred to another hospital, the local investigator will ensure compliance with the assigned treatment, data collection, and follow-up. If the patient does not attend scheduled follow-up, the investigator will telephone the participant and/or their general practitioner to identify any endpoints that may have occurred at home or at another hospital. At the time of randomization, study participants will be given stamped addressed envelopes with all questionnaires for secondary outcomes labelled with specific dates for completion to be mailed back to the local study investigator in case of inability to meet in person at scheduled follow-ups or in case the local investigator remains unsuccessful in contacting the patient by telephone.

### Data management {19}

Patient data will be collected and entered into the electronic case report form using REDcap by trained local study investigators or research nurses. In case of temporary unavailability of internet access, a paper version of the case report form is enclosed in the Trial Master File sent to each site before site initiation. The collected data in the paper version will then subsequently be entered into the electronic case report form by local site study personnel. Each site will also keep a screening log that will be sent by email to the Sponsor every 6 months using encrypted email.

The REDcap database is managed and secured by the IT-services of North Denmark Region. The Trial Master File and material containing patient information will be kept at local study sites at secured facilities and according to GDPR regulations. The quality of data of will be monitored online by the trial manager (JB) and onsite by GCP personnel.

### Confidentiality {27}

All personal data are entered and maintained within the REDcap tool hosted by North Denmark Region. Only authorized trial personnel including local study investigators, members of the Steering Committee, and the Data Monitoring and Safety Committee (DMSC) can access the stored information using personal usernames and passwords.

### Plans for collection, laboratory evaluation, and storage of biological specimens for genetic or molecular analysis in this trial/future use {33}

Blood samples will be collected at randomization and after 6 months. Each sampling will consist of:

1. 10 mL serum glass with gel (2 glasses of 5 mL each) which should be centrifuged for 10 min at 800×*g* (rcf) and then stored locally within 3–4 h at − 20 °C or colder until shipment.
2. 10 mL EDTA glass with gel (2 glasses of 5 mL each) which should be centrifuged for 10 min at 800×*g* (rcf) and then stored locally at − 20 °C or colder until shipment.
3. 6.0 mL EDTA glass which should be stored locally at − 20 °C or colder until shipment.

Using “freezer-proof” labels and pen, each glass will be labelled with the study record identification of the patient, date of sampling, and study site. Blood samples will be sent to the Department of Infectious Diseases at Aalborg University Hospital every 12 months and stored at − 80 °C in a research-biobank until analysis according to the specified substudy. After conclusion of the (sub)study, the blood samples will be stored in a biobank for an additional 10 years at conditions specified by the Danish data protection legislation in case new technology allows for new and more advanced immune-genetic analyses. At end of storage, the samples will be sent for destruction at a facility for biological waste management.

## Statistical methods

### Statistical methods for primary and secondary outcomes {20a}

Statisticians will remain blinded to randomization group. Concordant with CONSORT and SPIRIT guidelines, a flowchart will be used to describe patient recruitment including information of reasons for study exclusion. Baseline demographics will be presented stratified on treatment group (age, sex, predisposing conditions, comorbidities, symptoms and signs at time of randomization, blood tests, microbiological findings, and results of cranial imaging). Categorical variables will be reported as frequencies and percentages (based on non-missing sample size) along with missing values and compared by Fisher’s exact test. Continuous variables will be summarized with *n* (non-missing sample size), means with standard deviations or medians with interquartile rates and compared by Student’s *t* test (performed with bootstrap in case of non-normally distributed data).

Missing observations in primary outcome (and occurrence of each component of the composite primary endpoint) will be handled using pseudo-observations, worst-case scenario (i.e., all missing values in early switch to oral antibiotics are categorized as unfavorable while missing values for standard treatment are assigned a favorable outcome), and complete-case scenario [41].

The intention-to-treat population includes all randomized patients in the groups to which they were randomly assigned, regardless of compliance with the entry criteria, the treatment they actually received, withdrawal from treatment, or deviation from the protocol [42].

The per-protocol population in our study is defined as patients receiving at least 14 days of treatment assigned by randomization (to account for cross-over and non-adherence).

#### Primary analyses

In this non-inferiority study, we will first use the intention-to-treat population to examine the absolute risk difference of the primary outcome (binary endpoint) in the two randomized groups at 6 months after study inclusion. We will consider non-inferiority to be shown if the 95% CI excludes a treatment difference larger than 10% in favor of standard IV treatment.

We will illustrate occurrence of the primary endpoint and all its components by cumulative incidence plots and use Cox regression for analyses of primary and secondary endpoints adjusted for immunocompromise (diabetes mellitus, known alcohol abuse, asplenia, HIV/AIDS, solid cancer, prednisolone < 20 mg/day), level of comorbidity (0, 1–2, and ≥ 3), and congenital heart disease.

In case multiple patients are lost to follow-up, analyses of primary outcome will be performed using pseudo-observations.^37^

If non-inferiority is proven in the early switch to oral antibiotics arm, we will examine if early switch to oral treatment is superior to standard IV treatment using the same primary composite endpoint (*α* = 0.05 and two-sided 95% CI).

#### Secondary outcome analyses

Continuous endpoints (QoL scores and cognitive evaluations) obtained at randomization, end of treatment, and 3, 6, and 12 months since randomization will be analyzed using a repeated measures regression model, hereby taking the within-patient correlation into account. A global test for differences between randomization arms in the time-course will be performed, as well as pointwise comparisons. The analyses will be illustrated graphically with plots of mean and standard error over time by treatment arm.

Unfavorable outcome stratified by level of pre-existing comorbidity at time of randomization will be defined using E-GOS scores at end of treatment, and at 3, 6, and 12 months since randomization. Binary outcomes such as unfavorable outcome and all-cause mortality will be analyzed as above using mixed models. The risks will then be compared between groups taking the within-patient correlation into account. Crude as well as adjusted risks will be estimated. Confounder adjustments will include immunocompromise (diabetes mellitus, known alcohol abuse, asplenia, HIV/AIDS, solid cancer, prednisolone < 20 mg/day) and congenital heart disease.

The remaining secondary endpoints will be presented with mean and standard deviation or number and percentage and compared, when appropriate, by unpaired *t* test or Fisher’s exact test.

#### Sensitivity analyses

In sensitivity analyses, the intention to treat and the per-protocol analyses for the primary outcome and each component of the composite primary endpoint after 6 months since randomization, will be stratified in four strata by pathogen (oral cavity bacteria yes/no) and largest brain abscess diameter (± 3 cm).

Further, each component of the primary composite outcome will be compared using complete cases and worst-case scenarios.

Sensitivity analyses for the remaining secondary endpoints will not be performed.

### Interim analyses {21b}

Based upon the hypothesis of non-inferiority, we expect the primary outcome to occur in 44 patients (11% of 450 patients) during the trial with an equal distribution in both treatment arms. Interim analyses will be scheduled to be performed once 25 and 50% of the expected primary outcomes have occurred in either arm or in both arms combined, whichever occurs first [43]. The DMSC may recommend early termination of the ORAL trial if the *p* value is < 0.0110 for inferiority of early transition to oral therapy versus non-inferiority at the 10% margin after the occurrence of 25 or 50% of the expected primary outcome events [44].

### Methods for additional analyses (e.g., subgroup analyses) {20b}

#### Supplementary analyses

Subgroup analyses for the primary endpoint, each component of the primary composite outcome, and E-GOS will be displayed by a forest plot according to number of comorbidities (0/1–2/≥3), neurosurgical aspiration/excision of brain abscess (yes/no), age (± 65 years), oral cavity bacterial etiology (yes/no), IVROBA (yes/no), size of brain abscess (± 3 cm), number of brain abscesses (± 2), moderate immunocompromise (yes/no), and congenital heart disease (yes/no).

### Methods in analysis to handle protocol non-adherence and any statistical methods to handle missing data {20c}

Patients who withdraw from the randomized treatment will still have their primary outcome assessed after 6 months since randomization and will be included in the intention-to-treat analyses. Patients lost to follow-up will be censored at last known time to be alive. For all these analyses, we will follow patients from randomization until death, other components in the primary endpoint at 6 months since randomization or censoring, whichever comes first.

### Plans to give access to the full protocol, participant-level data, and statistical code {31c}

Beginning 6 months and ending 3 years after publication, an anonymized dataset, full study protocol, statistical analysis plan, informed consent form, clinical study report, and analytic code can be shared with qualified researchers who provide a methodologically sound proposal for a post hoc study assessed by the members of the Steering Committee. To gain access, data requestors will need to sign a data access agreement.

## Oversight and monitoring

### Composition of the coordinating center and trial steering committee {5d}

The steering committee consists of:

- Jacob Bodilsen (Aalborg University Hospital, Denmark)*
- Henrik Nielsen (Aalborg University Hospital, Denmark)*
- Matthijs C. Brouwer (Amsterdam UMC, The Netherlands)
- Diederik van de Beek (Amsterdam UMC, The Netherlands)
- Pierre Tattevin (Rennes University Hospital, France)
- Steven Tong (Royal Melbourne Hospital, Australia)
- Pontus Naucler (Karolinska, Stockholm, Sweden)

*Coordinating center.

The Sponsor is Henrik Nielsen. The initiative for the ORAL study has been taken by Jacob Bodilsen and Henrik Nielsen. Public involvement group or patient representatives have not been included in the design or conduct of the ORAL trial.

The steering committee (JB, HN, MCB, DvdB, ST, PN, PT) will be responsible for trial completion, data analysis, and interpretation. The steering committee will have the authority and responsibility to terminate the study, e.g., by recommendation from the Data Monitoring and Safety Committee. The steering committee, endpoint committee members, and local investigators will meet every 6 months either by telephone or at meetings to discuss study progression.

The coordinating center (Aalborg University Hospital) is responsible for the overall organization of the trial, day-to-day management of the trial, and coordination of the trial with the national coordinators. In turn, the national coordinators will assist the sponsor in obtaining government approvals and setting up sites within each country. Jacob Bodilsen is the primary investigator and trial manager assisted by a full-time research nurse.

All study investigators will be involved in critical review of the manuscript and acceptance of submission for publication of the final draft.

The blinded endpoint adjudication committee including a blinded neuroradiologist will determine the primary outcome by review of medical records and brain imaging redacted for information on allocated treatment and IV catheter insertion.

The Data Monitoring and Safety Committee will oversee safety aspects of the study and advice for early termination in case of futility, evident superiority of one treatment over the other, or serious safety concerns related to treatment.

### Composition of the data monitoring committee, its role and reporting structure {21a}

An independent Data Monitoring and Safety Committée (DMSC) consisting of two infectious diseases specialists and a clinical epidemiologist will oversee the study. They are required to have no competing interests. Interim analyses will be performed once 25 and 50% of the expected primary outcomes have occurred in either or both arms combined. However, the DMSC may also conduct unplanned interim analyses at their discretion. Results of interim analyses will not be available outside the DMSC unless pre-specified stopping rules are fulfilled, in which case the Steering Committee will be notified. The mandate and responsibility to terminate the study early lies within the Steering Committee. For further details, please see Appendix.

### Adverse event reporting and harms {22}

We will include electronic registration of date of onset, severity, and resolution of all potential expected SAEs and SUSARs along with an assessment of causality with the intervention by the local investigator. SUSARs will be reported according to the local requirements and regulations and to the European Union Directive 2001/20/EC from April 4, 2001 (Official Journal of the European Communities. 2001;121:34-44).

### Frequency and plans for auditing trial conduct {23}

Audits of trial conduct is not deemed possible due to the rarity of the disease at each study center, but experiences in study inclusion will be shared at meetings twice a year (teleconferences or physical meetings).

### Plans for communicating important protocol amendments to relevant parties (e.g., trial participants, ethical committees) {25}

Important protocol modifications (e.g., changes in inclusion criteria, outcomes, or statistical analyses) will be disseminated to the Ethics Committees or institutional review boards, national competent authorities including medicines agencies, trial registries, and local study investigators. Furthermore, published trial protocols will be updated.

### Dissemination plans {31a}

Regardless of outcome, the results of the primary study will be made available to the public, preferably by publication in a high-impact international, peer-reviewed medical journal with an open access format.

## Discussion

Early oral antibiotic therapy may lead to reduced compliance and may be inferior to standard IV treatment. On the other hand, if non-inferiority of early switch to oral antibiotic therapy is proven, it may have a substantial impact on patients’ lives and assist in alleviating psychological stress during treatment and, for some patients, offer the benefit of treatment in the comfort of their own homes. It may also reduce physical risks associated with long-term hospitalization (e.g., hospital-acquired infections and loss of activities of daily life functions) and IV treatment (bleeding, infection, venous thrombosis, or need for catheter replacement). The potential of up to a 75% reduction of admission time from 6 or 8 weeks to 2 weeks is naturally also of interests for both patients and health care services not only in economically privileged countries, but also in settings where patients must pay for treatment themselves or where health care resources are limited.

## Trial status

ORAL study protocol version 1.2 dated March 10, 2021. The first center opened for inclusion on November 3, 2020 (Aalborg, Denmark). Expected trial duration is 4 years (3 years of patient inclusion + 1 year of follow-up). The study is expected to open in Sweden, France, the Netherlands, and Australia during 2021 and 2022 with delays attributable to the COVID-19 pandemic.

## Appendix

**Charter for the Data Monitoring and Safety Committee (DMSC)**

### Introduction

The DMSC will constitute its own plan of monitoring and meetings. However, this charter will define the minimum of obligations and primary responsibilities of the DMSC as perceived by the Steering Committee, its relationship with other trial components, its membership, and the purpose and timing of its meetings. The charter will also outline the procedures for ensuring the confidentiality and proper communication, the statistical monitoring guidelines to be implemented by the DMSC, and an outline of the content of the open and closed reports which will be provided to the DMSC.

*Primary responsibilities of the DMSC*

The DMSC will be responsible for safeguarding the interests of trial patients, assessing the safety and efficacy of the interventions during the trial, and for monitoring the overall conduct of the clinical trial. The DMSC will provide recommendations about stopping or continuing the trial to the Steering Committee of the ORAL trial. To contribute to enhancing the integrity of the trial, the DMSC may also formulate recommendations relating to the selection and recruitment of patients, their management, improving adherence to the regimens specified by the protocol and retention of patients, and the procedures for data management and quality control.

The DMSC will be advisory to the Steering Committee. The Steering Committee will be responsible for promptly reviewing the DMSC recommendations, to decide whether to continue or terminate the trial, and to determine whether amendments to the protocol or changes in trial conduct are required.

The DMSC is planned by protocol to meet physically in order to evaluate the planned interim analyses of the ORAL trial. The interim analyses will be performed by an independent statistician, who could conveniently also be the biostatistician sitting in the DMSC, selected by the members of the DMSC. The DMSC may additionally meet whenever they decide or contact each other by telephone or email in order to discuss the safety for trial participants. The sponsor has the responsibility to report the overall number of SAEs yearly to the DMSC. The DMSC can, at any time during the trial, request data on the distribution of events, including outcome measures and SAEs according to the intervention groups. Further, the DMSC can request unblinding of the interventions if suggested by the data, see section “Closed sessions.” The recommendations of the DMSC regarding stopping, continuing or changing the design of the trial should be communicated without delay to the Steering Committee of the ORAL trial. As fast as possible, and no later than 48 h, the Steering Committee has the responsibility to inform all study investigators and sites about the recommendation of the DMSC and the Steering Committees decision hereof.

*Members of the DMSC*

The DMSC is an independent multidisciplinary group consisting of clinicians and a biostatistician that, collectively, has experience in the management of brain abscess patients and in the conduct, monitoring, and analysis of randomized clinical trials.

DMSC Members

- Professor Peter Skinhøj, Copenhagen University, Denmark
- Professor Thomas Benfield, Department of Infectious Diseases, Hvidovre Hospital, Denmark
- Professor Mette Nørgaard, Department of Clinical Epidemiology, Aarhus University Hospital, Denmark

DMSC Biostatistician

To be determined

### Conflicts of interest

DMSC members are required to be free of conflicts of interests and sign a declaration thereof (Appendix 13). Such sources of conflicts may be financial, scientific, or regulatory in nature. Thus, neither trial investigators nor individuals employed by the sponsor, nor individuals who might have regulatory responsibilities for the trial products, are to be members of the DMSC. The DMSC members may not own stock in the companies having products being evaluated by the ORAL trial. The DMSC members will disclose to fellow members any consulting agreements or financial interests they may have with sponsors or products used in the trial, with the contract research organization (CRO) for the trial (if any), or with sponsors having products that are being evaluated or having products that are competitive with those being evaluated in the trial. The DMSC will be responsible for deciding whether those consulting agreements or financial interests may jeopardize their objectivity. The DMSC members will be responsible for advising fellow members of any changes in these consulting agreements and financial interests that occur during the course of the trial. Any DMSC members who develop significant conflicts of interests during the course of the trial should resign from the committee. DMSC membership is to be for the duration of the clinical trial. If any members leave the DSC during the course of the trial, the Steering Committee will appoint the replacement(s).

*Formal interim analyses meeting*

Interim analysis meetings will be held to review data relating to treatment efficacy, patient safety, and quality of trial conduct. The three members of the DMSC will meet when 6-month follow-up of 100 and 200 (approximately 50 % of sample size estimation) patients have been obtained.

*Proper communication*

To enhance the integrity and credibility of the trial, procedures will be implemented to ensure that the DMSC has sole access to evolving information from the clinical trial regarding comparative results of efficacy and safety data aggregated by treatment group. At the same time, procedures will be implemented to ensure that proper communication is achieved between the DMSC and the trial investigators. To provide a forum for exchange of information among various parties who share the responsibility for the successful conduct of the trial, a format for open sessions and closed sessions will be implemented. The intent of this format is to enable the DMSC to preserve confidentiality of the comparative efficacy result, while at the same time providing opportunities for interaction between the DMSC and others who have valuable insights into trial-related issues.

*Closed sessions*

Sessions involving only DMSC members (called closed sessions) will be held to allow discussion of confidential data from the clinical trial, including information about the relative efficacy and safety of interventions. In order to ensure that the DMSC will be fully informed in its primary mission of safeguarding the interest of participating patients, the DMSC will be blinded in its assessment of safety and efficacy data. However, the DMSC can request unblinding from the Steering Committee.

Closed reports will include analysis of the primary outcome measure. In addition, analyses of the secondary outcome measures and SAEs will also be reported. These closed reports will be prepared by the independent biostatistician member of the DMSC with assistance from the trial data manager in a manner that allows them to remain blinded. The closed reports should provide information that is accurate and with complete follow-up of the primary endpoint (including all-cause mortality) within 2 months of the date of the DMSC meeting.

*Open reports*

For each DMSC meeting, open reports will be provided to all who attend the DMSC meeting. The reports will include data on recruitment, baseline characteristics, pooled data on eligibility violations, completeness of follow-up, and compliance. The independent statistician being a member of the DMSC will prepare these open reports in co-operation with the trial data manager. The reports should be sent to the DMSC members approximately 3 days prior to the date of the meeting.

*Minutes of the DMSC Meetings*

The DMSC will prepare minutes of their meetings. The closed minutes will describe the proceedings from all sessions of the DMSC meeting, including the listing of recommendations by the committee. Because it is possible that these minutes may contain unblinded information, it is important that they are not made available to anyone outside the DMSC.

*Recommendations to the Steering Committee*

After the interim analysis meetings, the DMSC will make a recommendation to the Steering Committee to continue, hold, or terminate the trial. This recommendation will be based primarily on safety and efficacy considerations and will be guided by statistical monitoring guidelines defined in this charter and the trial protocol.

Common methods for interim analyses of superiority trials such as Haybittle-Peto or Lan-deMets stopping criteria, often combined with statistical boundaries based on O’Brien-Fleming alfa-spending function, may be too conservative for use in non-inferiority trials [43]. Consequently, interim analyses in the ORAL trial will be performed in accordance with the principles of Earliest Information Time and using boundaries more appropriate for non-inferiority trials [43, 44]. Compared with standard methods, large reductions in exposed patients is possible using this approach in case one treatment arm is unexpectedly inferior to the other, although there may be a slight loss of power compared with standard stopping criteria (< 1%) [43].

Based upon the hypothesis of non-inferiority, we expect the primary outcome to occur in 44 patients (11% of 400 patients) during the trial with an equal distribution in both treatment arms. Interim analyses will be scheduled to be performed once 25 and 50% of the expected primary outcomes have occurred in either arm or in both arms combined, whichever occurs first [43]. The DMSC may recommend early termination of the ORAL trial if the *p* value is < 0.0110 for inferiority of early transition to oral therapy versus non-inferiority at the 10% margin after the occurrence of 25 or 50% of the expected primary outcome events [44]. If interim analyses show non-inferiority of the experimental arm, the study should be allowed to continue due to the subjectivity of the non-inferiority margin and to ensure statistical certainty of the final results. In case of superiority of early switch to oral antibiotics, the study will be allowed to continue unless oral antibiotic therapy is shown to be superior to standard treatment by 20% or more with a *p* value < 0.05.

The Steering Committee is jointly responsible with the DMSC for safeguarding the interests of participating patients and for the conduct of the trial. Recommendations to amend the protocol or conduct of the trial made by the DMSC will be considered and either accepted or rejected by the Steering Committee. The Steering Committee will be responsible for deciding whether to continue, hold, or stop the trial based on the DMSC recommendations. Any substantial changes to the study protocol or conduct of the trial will be consulted with the DMSC prior to their implementation.

*Statistical monitoring guidelines*

The outcome parameters are defined in the statistical analyses plan in the protocol. For the two intervention groups, the DMSC will evaluate data on:

- The primary outcome measure consisting of 6-month risks of all-cause mortality, IVROBA, unplanned (re-)aspiration, or excision of brain abscess, relapse, or recurrence

In addition, each component will be analyzed independently after end of treatment, 3, 6, and 12 months of follow-up since randomization:

- All-cause mortality
- IVROBA
- Unplanned (re-)aspiration or excision of brain abscess
- Relapse (overall and by relapse subgroups a, b, and c)
- Recurrence

The DMSC will be provided with these data from the coordinating study center (Aalborg) as:

- Number of patients randomized
- Number of patients randomized per intervention group
- Number of patients per stratification variable in each intervention group
- Number of outcome events (primary and secondary) in the two groups

Based on evaluations of these outcomes, the DMSC will decide if they want further data from the coordinating center and when to perform the next analysis of the data.

For analyses, the data will be provided in one file as described below. If protocol specified event rates are inaccurate, the DMSC may also be asked to ensure that procedures are properly implemented in order to adjust the trial sample size or duration of follow-up to restore power. If so, the algorithm for doing this should be clearly specified.

The DMSC will be provided with a file containing the data defined as follows:

- Row 1 contains the names of the variables (to be defined below).
- Row 2 to *N* (where *N* − 1 is the number of patients having entered the trial) each contains the data of the individual patients
- Column 1 to *p* (where *p* is the number of variables to be defined below) each contains in row 1 the name of a variable and in the next *N* rows the values of this variable.

The values of the following variables should be included in the database:

1. Screening id: A number that uniquely identifies the patient
2. Rand code: The randomization code (Group 0 or 1). The DMSC is not to be informed on what interventions the groups received
3. The primary composite endpoint (1 = Yes, 0 = No)
4. All-cause mortality (1 = Yes, 0 = No)
5. IVROBA (1 = Yes, 0 = No)
6. Unplanned (re-)aspiration or excision of brain abscess (1 = Yes, 0 = No)
7. Relapse including by subgroups a, b, and c (1 = Yes, 0 = No)
8. Recurrence (1 = Yes, 0 = No)

## Abbreviations

AE: Adverse event
AIDS: Acquired immunodeficiency syndrome
CCI: Charlson Comorbidity Index
CI: Confidence interval
CNS: Central nervous system
DASGIB: Danish group of infections of the brain
DMSC: Data monitoring and safety committee
ESGIB: European study group of infections of the brain
EudraCT: European Union Drug Regulating Authorities Clinical Trials
E-GOS: Extended Glasgow outcome scale
GCP: Good clinical practice
GOS: Glasgow outcome scale
HIV: Human immunodeficiency virus
IV: Intravenous
IVROBA: Intraventricular rupture of brain abscess
SAE: Serious adverse event
SUSAR: Suspected unexpected serious adverse reaction
